# Book review

**DOI:** 10.1155/S1463924691000457

**Published:** 1991

**Authors:** Peter B. Stockwell


### Book review

**Supercritical Fluid Chromatography**
(RSC Chromatography Monographs). Edited by ROGER M. SMITH (Cambridge: Royal Society of Chemistry.) ISBN 0-85186-577-1

*Supercritical Fluid Chromatography* is an ideal topic to launch the new RSC Chromatographic Monograph series. The book has been derived from a comprehensive short course run by the Analytical Research Group at Loughborough University and is edited by Roger Smith. The papers concentrate primarily on methods employed in SPC separations and the chromatographs ability to control these. SFC provides a wide range of separations from gas like separators similar to GLC to liquid separators similar to HPLC, it has been said that SFC offers the best of both worlds.

The book is well presented and is well recommended as an introduction to the subject area. Its chapters relate to the theory and principles of SPC, the Instrumentation available for SFC and the selection of conditions for use. Coupling to techniques such as mass spectrometry are also dealt with in detail. The price of the book is £27·50 which in today's economic environment is extremely good value for money.

**Peter B. Stockwell**
PS Analytical Ltd

### Erratum

*Journal of Automatic Chemistry*, Vol. 13, No. 2, March–April 1991

The ISBN for the first book review on p. 81 of the above issue is incorrect. The correct details are as follows:

**An Introduction to Laboratory Automation.** By VICTOR CERDÁ and GUILLERMO RAMIS (John Wiley & Sons Ltd, Chichester, 1990). ISBN 471-61818-7.

